# PTBP3 regulates proliferation of lung squamous cell carcinoma cells via CDC25A‐mediated cell cycle progression

**DOI:** 10.1186/s12935-022-02448-7

**Published:** 2022-01-11

**Authors:** Yingji Chen, Ying Ji, Suo Liu, Yicai Liu, Wei Feng, Longyu Jin

**Affiliations:** 1 Department of Cardiothoracic Surgery, Third Xiangya Hospital of Central South University, 138 Tongzipo Road, Changsha City, Hunan China; 2 Department of Thoracic Surgery, Beijing Chao-Yang Hospital, Capital Medical University, Beijing, 100020 China

**Keywords:** PTBP3, LUSC, Proliferation, CDC25A, Cell cycle

## Abstract

**Background:**

The roles of Polypyrimidine tract-binding protein 3 (PTBP3) in regulating lung squamous cell carcinoma (LUSC) cells progression is unclear. The aim of this study was to investigate the role of PTBP3 in LUSC.

**Methods:**

Expression and survival analysis of PTBP3 was firstly investigated using TCGA datasets. Quantitative reverse transcription PCR and Western blot were performed to detect PTBP3 expression in clinical samples. Moreover, cell counting kit 8 (CCK-8) assays, colony formation assays and in vivo tumor formation assays were used to examine the effects of PTBP3 on LUSC cell proliferation. RNA-sequence and analysis explores pathways regulated by PTBP3.Flow cytology was used analyzed cell cycle. Cell cycle-related markers were analyzed by Western blot.

**Results:**

PTBP3 was found to be overexpressed in LUSC tissues compared with normal tissues. High PTBP3 expression was significantly correlated with poor prognosis. In vitro and vivo experiments demonstrated that PTBP3 knockdown caused a significant decrease in the proliferation rate of cells. Bioinformatics analysis showed that PTBP3 involved in cell cycle pathway regulation in LUSC. Furthermore, PTBP3 knockdown arrested cell cycle progression at S phase via decreasing CDK2/Cyclin A2 complex. In addition, downregulation of PTBP3 significantly decreased the expression of CDC25A.

**Conclusions:**

Our results suggest that PTBP3 regulated LUSC cell proliferation via cell cycle and might be a potential target for molecular therapy of LUSC.

**Supplementary Information:**

The online version contains supplementary material available at 10.1186/s12935-022-02448-7.

## Introduction

Lung cancer remains the leading cause of cancer mortality in the worldwide [1, 2]. Non-small cell lung cancer (NSCLC) accounts for 80% of lung cancer cases [3], of which lung squamous cell carcinoma (LUSC) and lung adenocarcinoma (LUAD) are the major subtypes, and lung squamous cell carcinoma (LUSC) rank the second common form of NSCLC [4]. Despite great advances in LUSC treatment, the long-term survival of patients with LUSC is still poor [5]. Thus, more research into the mechanisms and molecular functions of oncogenes is urgently needed to help identify new therapeutic targets.

Uncontrolled cell proliferation is the hallmark of cancer [6, 7]. Accordingly, suppressing the proliferation of cancer cells play an important role in anticancer treatment. In eukaryotic cells, proliferation is primarily regulated by cell cycle [8]. Proper progression throughout the cell division cycle depends on the expression level of a family of proteins known as cyclins, and the subsequent activation of cyclin-dependent kinases (CDKs) [9]. Hence, blocking cell cycle related checkpoints by inhibiting oncogenes is an effective way to repress the proliferation of cancer cells.

PTBP3 is also named as regulator of differentiation 1 (ROD1), which is encodes an RNA binding protein [10]. PTBP3 plays a crucial role in regulating gene expression through a multitude of RNA-binding proteins that affect the biological behavior of carcinoma cells [11]. Previous study showed PTBP3 protein level was increased in colorectal cancer [12], breast cancer [13], and gastric carcinoma [14]. PTBP3 was also verified regulation of migration in NSCLC [2]. However, whether it plays an important role in the proliferation of LUSC remains to be determined.

In current study, we found that PTBP3 was markedly upregulated in LUAD and LUSC patients, but survival analysis suggested that a significant prognostic effect only in LUSC patients. We further confirmed that the expression of PTBP3 was also significantly increased in clinical samples of LUSC patients. Knockdown PTBP3 expression inhibited LUSC cell proliferation in cultured cell lines and decreased tumor growth in animal model. Importantly, we found that PTBP3 may regulate the cell cycle by regulating CDC25A protein expression. These data indicated that PTBP3 could be a potential biomarker and therapeutic target for LUSC.

## Methods and materials

### Tissue collection and ethics statement

Primary LUSC tissue were obtained from the Third Xiangya Hospital of Central South University. These patients had not received radiotherapy, chemotherapy, targeted therapy and immunotherapy before surgery. Appropriate ethical approval was obtained from the Third Xiangya Hospital Ethics Committee, and written informed consent was obtained from all patients. Fresh LUSC tumor tissues and their adjacent non-malignant lung tissues were sampled and stored at − 80 °C.

### Cell lines and cell culture

NCI-H520 and NCI-H1703 cell lines were obtained from ATCC (Gaithersburg, MD, USA). The cell lines were cultured in RIMP-1640(Gbico, USA) at 37 °C in a humidified atmosphere of 5% CO2. All the above mediums were supplemented with 10% fetal bovine serum (FBS, Gbico, USA).

### Lentivirus transduction

NCI-H520 and NCI-H1703 cells were seeded in six-well plates 24 h before transfection. When confluency reached 60–70%, lentivirus encoding PTBP3 shRNA or scrambled negative control (NC) shRNA purchased from Genepharma (Shanghai, China) was added at the multiplicity of infection recommended by the manufacture. To generate stable lentivirus-transduced lines, cells were infected with virus, and stable cell lines were selected with 2 µg/ml puromycin treatment after 72 h of transfection. Transfection efficiency of PTBP3 was assessed by quantitative real-time PCR (qRT-PCR) and western blot. All sequences are provided in Additional file 1: Table S1.

### Quantitative real-time PCR assays

Total RNA from cells and tissues was extracted using SteadyPure Universal RNA Extraction Ki according to the manufacturer’s instructions. (AG21017, China). Reverse transcription was performed using the qPCR RT Master Mix with gDNA Remover (Toyobo, Osaka, Japan) according to the manufacturer’s instructions. Quantitative real-time PCR (qRT-PCR) was carried out on a LightCycler 480 Real-Time PCR instrument (Roche, Basel, Switzerland) using SYBR Green Real-time PCR Master Mix (Toyobo). Analysis was performed using the 2^−∆∆Ct^ method, with GAPDH as the endogenous control. All primer pairs were purchased from Tsingke (Beijing, China), and all sequences are provided in Additional file 1: Table S1.

### Western blot analysis

Cells were separately harvested and lysed by RIPA buffer (CWbio, Beijing, China) containing 0.1 mg/mL PMSF (Keygen, Nanjing, China) and protease inhibitor (Roche, Man-nheim, Germany). The procedure of western blot was same as our previous research [15]. Antibodies against PTBP3, Cyclin A2 as well as goat anti-rabbit IgG-HRP antibodies and goat anti-mouse IgG-HRP were purchased from Proteintech (Wuhan, China). Antibodies against CDC25A, CDK2 were purchased from Wanleibio (China).

### Cell proliferation assay

Cell proliferation was assessed by CCK-8 and colony formation assays. For the CCK-8 assay, stably transfected cell lines seeded into 96-well plates at 1 × 10^3^ cells per well at a concentration of 100 μL, with five replicates for each condition. After incubation for an additional 0, 24, 48 or 72 h., 10μL CCK-8 reagents (Biosharp, China) were added to each well, and the cells were cultured at 37 °C for 2 h. The OD value was then measured at 450 nm using spectrophotometer. For colony formation assays, stably transfected cell lines seeded (1 × 10^3^ cells/well) into 6-well plate, After1-2 weeks of culture, the colonies were fixed methanol, stained with crystal violet and analyzed using ImageJ software.

### RNA-sequence

Total RNA was extracted using Trizol according to the manufacturer’s instructions. Libraries were constructed using NEBNext® Ultra™ RNA Library Prep Kit for Illumina® (NEB USA) and sequence run was performed on an Illumina Novaseq platform (USA). Differentially expressed genes (DEGs) were determined using a p value less than 5% and a fold change greater than 1.

### Bioinformatics analysis

From the TCGA database (https://portal.gdc.cancer.gov/) to download LUAD (Tumor:497 Normal:54) and LUSC (Tumor:502 Normal: 49) RNA-seq gene expression profile and survival information of patients. The “Limma” package of R software was used to process the RNA expression profile, and the repeated data were averaged. R package “SurvMiner” for survival analysis, analysis of OS in different risk groups. The receiver operating characteristic (ROC) curve was used to evaluate the accuracy of the PTBP3 in LUSC by comparing the area under the curves (AUCs). TCGA database through Spearman’s correlation analysis the relationship between PTBP3 and CDK2, cyclin A2, CDC25A in LUSC. A gene ontology (GO) including molecular function (MF), cellular components (CC) and biological processes (BP) and Kyoto Encyclopedia of Genes and Genomes (KEGG) analysis were both conducted using the Database for Annotation, Visualization and Integrated Discovery (DAVID), analysis tools for extracting meaningful biological information from multiple gene and protein collections. The data used for Gene set enrichment analysis (GSEA) were obtained from TCGA. The high and low groups of clinical LUSC specimens were separated according to the median PTBP3 expression level.

### Cell cycle analysis

Cells were fixed with 75% ethanol for 12-16 h, and stained with propidium iodide (PI, Sigma-Aldrich, USA) Percentage of cells in different cell cycle phases was analyzed using a FACS Caliber flow cytometer (Beckman USA).

### Immunohistochemistry (IHC) staining

IHC analysis was performed on tissue sections tumors from nude mouse model. All tissue specimens were formalin-fixed, embedded in paraffin, and then were deparaffined and rehydrated. Next, endogenous peroxidase activity was blocked by incubating the tissue sections with 3% hydrogen peroxide for 20 min, washed for 3 min three times with phosphate-buffered saline. After that, the tissue sections were blocked in 3% BSA for 30 min and incubated with anti-human Ki-67 (1:100) antibodies and anti-human PTBP3 antibody (1:100) at 4 °C overnight. The tumor sections were then incubated in biotinylated secondary antibodies for 50 min at room temperature, then developed with diaminobenzidine solution and counterstained with hematoxylin. Images were captured using an inverted microscope system (IX73; Olympus, Japan).

### In vivo tumor growth and immunohistochemistry assays

Male nude mice, 4–6 weeks old, were obtained from the Center for Medical Experiments of Third Xiangya Hospital of Central South University. The research protocol was approved, and the mice were maintained according to the Institutional Guidelines of the Animal Ethics Committee of Central South University. Mice were sacrificed by carbon dioxide asphyxiation. put the mouse into the euthanasia box and pour carbon dioxide into the box at a rate of about 40% of the replacement volume of the euthanasia box every minute, expose it for about 5 min, and confirm that the mouse is not moving, not breathing, and pupils are dilated. Turn off carbon dioxide and observe for another 2–3 min to confirm that the mouse is dead. Nude mice were randomized into two groups (3 mice/group) and injected subcutaneously separately with H520-sh-NC or H520-sh-PTBP3 cells (1 × 10^6^ cells/100 μl) in the left axilla. Tumor growth was monitored weekly from day 5 after injection using a standard caliper. Tumor volume (mm^3^) was calculated as: 1/2(length × width^2^), whereas length is the longest longitudinal diameter and width is the longest transverse diameter. Tumors were further embedded in paraffin for H&E and IHC.

### Statistical analysis

All statistical analyses were conducted using SPSS 26.0 (IBM USA). Data were shown as mean ± standard deviation (SD). Student’s t-test was used to analyze the assays. One-way analysis of variance (ANOVA) was used for comparison between the different groups. P value < 0.05 was considered statistically significant. For all statistics, data from at least three experiments were used.

## Results

### PTBP3 overexpression is associated with LUSC poor prognosis.

**Fig. 1:**
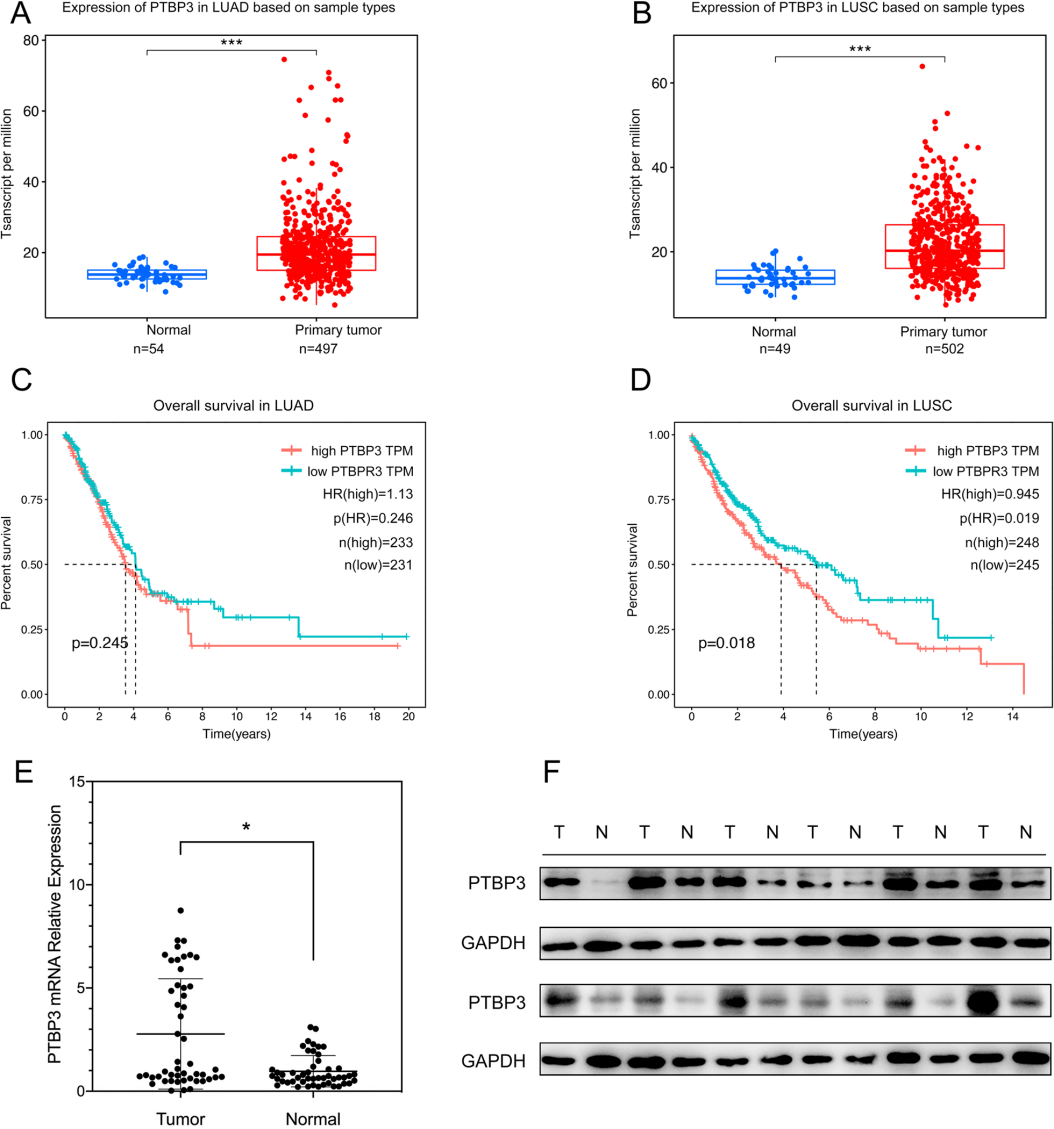
PTBP3 expression is significantly upregulated in LUSC and negatively correlated with the OS of LUSC patients. **A**, **B** The mRNA expression of PTBP3 in TCGA database including lung adenocarcinoma (LUAD) dataset and lung squamous cell carcinoma (LUSC) dataset. **C**, **D** The overall survival in LUAD and LUSC patients base on TCGA dataset. **E** PTBP3 mRNA level was detected in LUSC and normal tissues via qRT‐PCR assay. **F** PTBP3 protein level was examined in LUSC and normal tissues via Western blot assays. Data were presented as mean ± SD; *P < 0.05, ***P < 0.001

We first investigated the PTBP3 expression in LUAD (Fig. 1A) and LUSC (Fig. 1B), both of which were significantly increased in tumor tissues compare to normal tissues base on TCGA dataset. Survival analysis was performed to evaluate the impact of PTBP3 expression on the overall survival (OS) in patients with LUAD (Fig. 1C) and LUSC (Fig. 1D). Interestingly, in the survival analysis, LUSC patients showed a significant difference while LUAD patients did not. Besides, for the prediction of OS in LUSC patients, the 1,3,5-year AUC values of the ROC curve were higher than 0.5 (Additional file 2: Fig. S1), which showed a good survival prediction performance. We then detected PTBP3 expression in our own clinical specimens. qRT-PCR assay in 49 pairs of primary LUSC tissues and adjacent noncancerous tissues revealed higher PTBP3 mRNA level in LUSC tissues (Fig. 1E). We summarized the clinicopathological characteristics of these patients, and the data demonstrated that patients with higher PTBP3 expression showed a larger tumor size than the patients with lower PTBP3 expression (Table 1). Such result was observed in Western blot analyses based on 12 pairs of LUSC tissues and adjacent noncancerous tissues (Fig. 1F).

**Table 1:** Clinic-pathological characteristics of enrolled patients

Clinicopathological characteristics	Total (n = 49)	PTBP3		P-value
		High^a^ (n = 13)	Low^a^ (n = 36)	
Age				0.682
≤ 60	25	6	19	
> 60	24	7	17	
Gender				0.742
Male	31	9	22	
Female	18	4	14	
Differentiation				0.449
Low	22	7	15	
Moderate and High	27	6	21	
Tumor size				0.029*
> 3 cm	25	10	15	
≤ 3 cm	24	3	21	
Lymph node metastasis				0.39
Positive	20	4	16	
Negative	29	9	20	
Distant metastasis				–
M0	49	13	36	
M1	0	0	0	

^a^High and low expression groups were determined by the cutoff-point 25% (13 of 49) and 75% (36 of 49) of PTBP3 in 49 tumor tissue specimens

*Statistical significance (P < 0.05)

### PTBP3 knockdown inhibits the proliferation of LUSC cells in vitro and in vivo

**Fig. 2:**
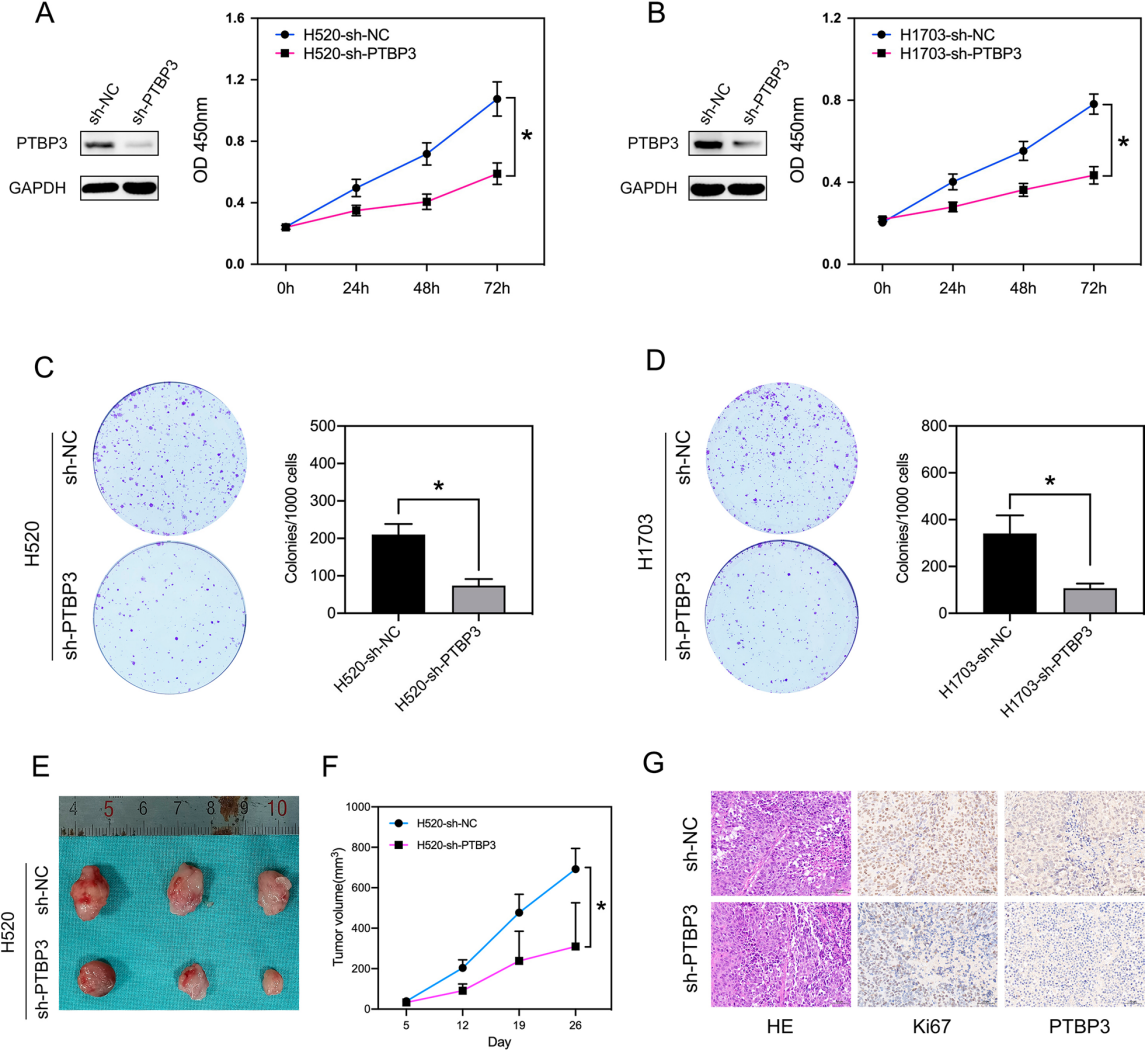
Effects of PTBP3 on tumor growth in cultured cells and an animal model of LUSC. **A**, **B** CCK-8 assay showing the proliferation ability of the H520 and H1703 cells with PTBP3 knockdown. **C**, **D** Clone formation assay showing the proliferation ability of the H520 and H1703 cells with PTBP3 knockdown. **E**, **F** Effect of PTBP3 knockdown in H520 cells on the xenograft model was assessed by evaluating tumor volume. **G** IHC detection of PTBP3, Ki67 in xenograft tumors formed by H520. Scale bar: 50 μm. Data were presented as mean ± SD; *P < 0.05

We next construct lentiviral shRNA specific for PTBP3. qRT-PCR and Western blot analyses revealed significantly reduced PTBP3 mRNA and protein level in H520 cells and H1703 cells. CCK-8 analysis showed that knockdown of PTBP3 significantly reduced proliferation of H520 (Fig. 2A) and H1703 (Fig. 2B) cells. Colony formation assays also showed the colony-forming ability was dramatically decreased in H520 (Fig. 2C) and H1703 (Fig. 2D) cells infected with sh-PTBP3.

To examine if PTBP3 promotes tumor growth in vivo, we selected nude mice for subcutaneous injection of H520 cells transduced with either sh-NC or sh-PTBP3 lentivirus. H529 cells xenograft transduced with sh-PTBP3 grew at a lower rate than the control (Fig. 2E, F). IHC showed that PTBP3 knockdown significantly decreased Ki67 expression in xenograft tumor (Fig. 2G).

### PTBP3 knockdown inhibits the proliferation of cells by regulating cell cycle

**Fig. 3:**
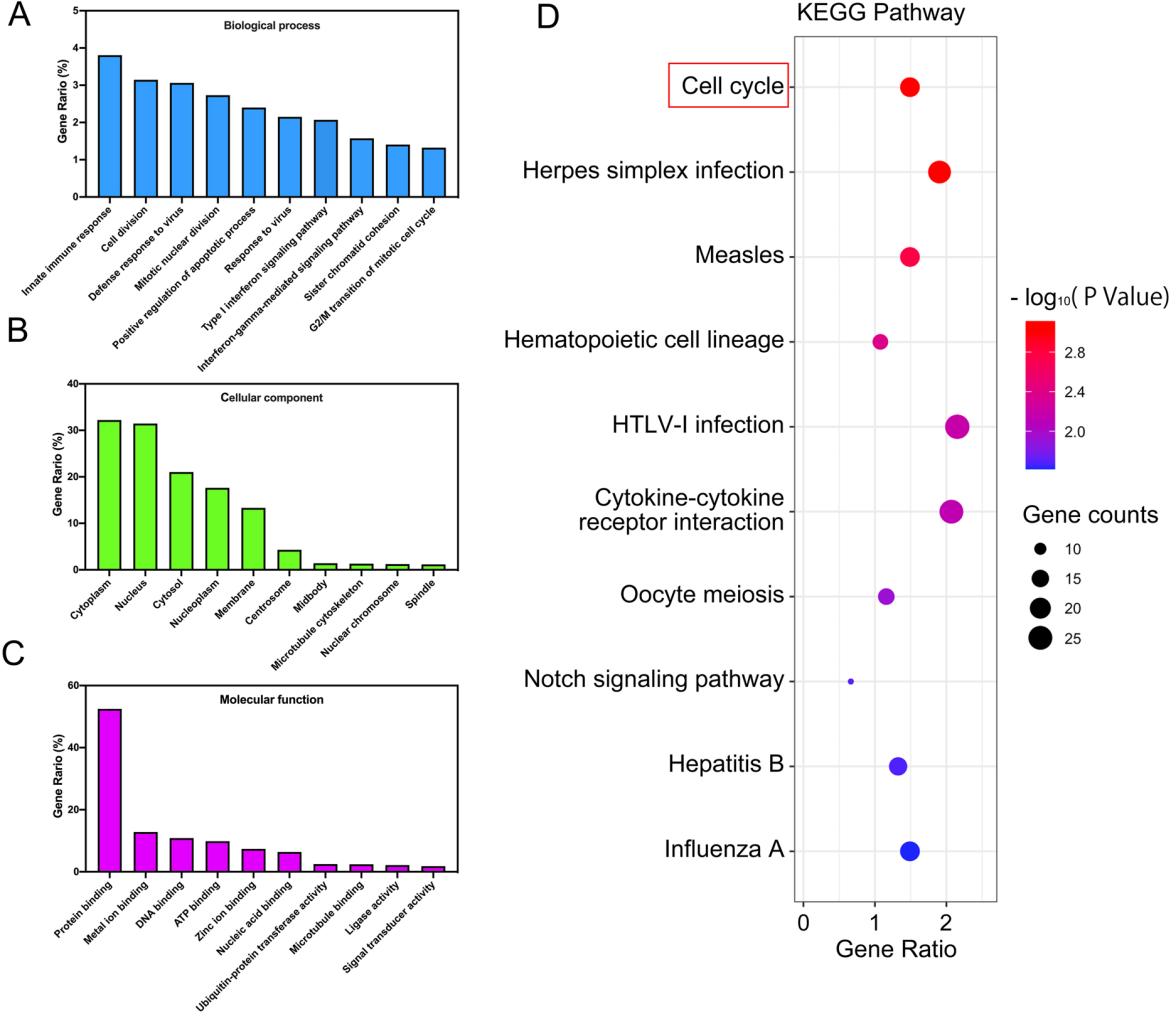
Bioinformatic analysis. **A**–**C** GO enrichment analysis with all DEGs **D** KEGG Pathway analysis with DEGs in RNA-seq

To gain a comprehensive understanding of the biological processes and molecular functions regulated by PTBP3, RNA-sequence was performed using H520 cells with or without PTBP3 knockdown. DEGs were applied with heat map (Additional file 3: Fig. S2A) and GO terms (Fig. 3A–C) and simultaneously, pathway analysis (Fig. 3D) was performed based on the KEGG database. These GO terms and enriched pathways suggest that PTBP3 regulates a subset of genes involved in cell division processes, such as cell cycle regulating, besides, GSEA analysis base on TCGA LUSC dataset showed a strong correlation between PTBP3 high expression and the cell cycle pathway (Additional file 3: Fig. S2B, C).

### PTBP3 knockdown leads to S phase cell cycle arrest

**Fig. 4:**
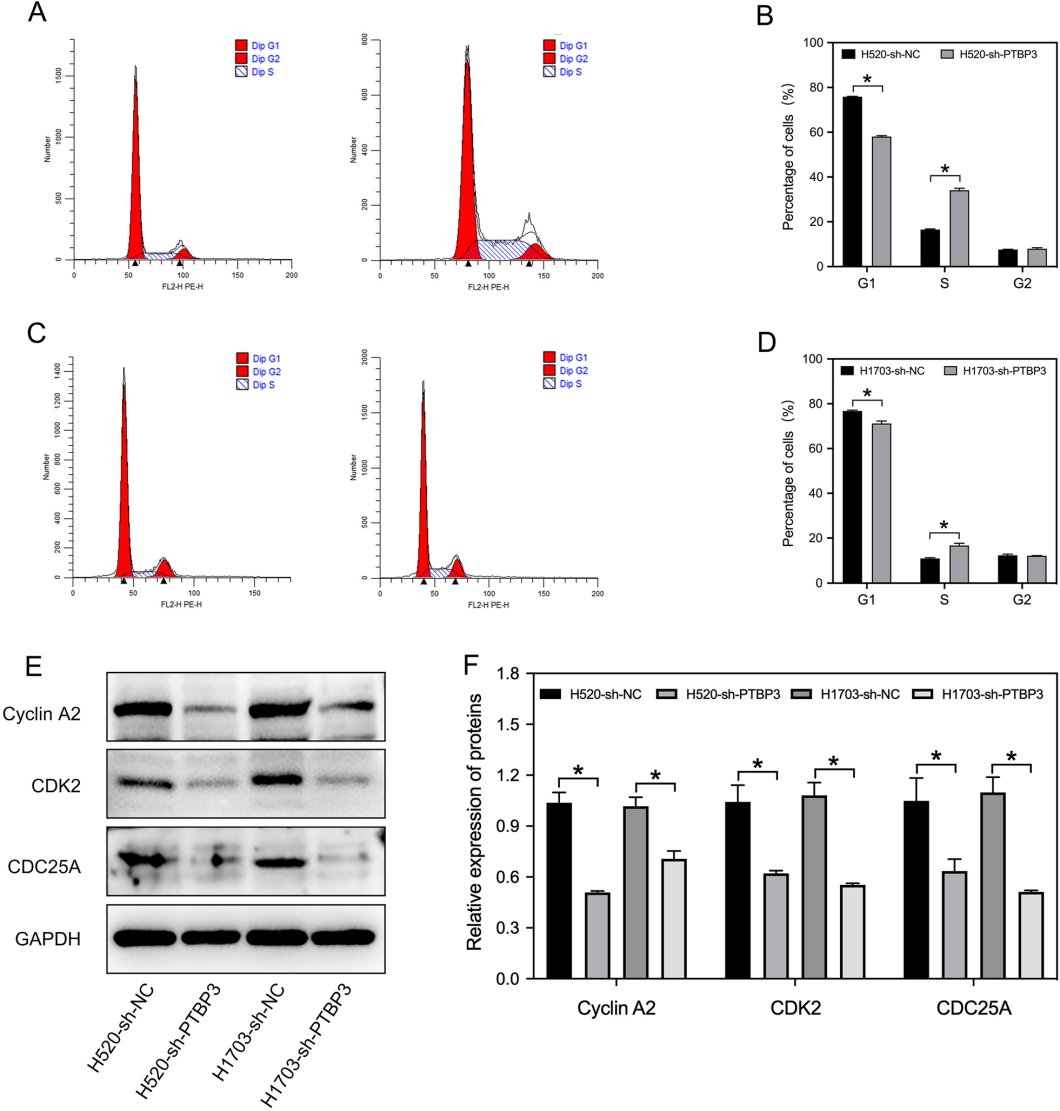
PTBP3 knockdown induces cell cycle arrest. **A**–**D** Cell cycle distribution was determined by flow cytometric analysis in H520 and H1703 cells after PTBP3 knockdown. The percentage of cells in the G1, S, and G2 phases was calculated. **E**, **F** Western blot analysis of proteins associated with cell cycle regulation in H520 and H1703 cells after PTBP3 knockdown. The integrated band density was determined using ImageJ Software, and GAPDH as the internal control. Data were presented as mean ± SD; *P < 0.05

To further understand the relationship between PTBP3 and cell cycle, Flow cytometry was used to evaluate the effect of PTBP3 on cell cycle changes. Knockdown of PTBP3 significantly increased the percentage of S phase H520 (Fig. 4A, B) and H1703 (Fig. 4C, D) cells compared to the control group. The expression of S phase related proteins Cyclin A2 and CDK2 was decreased by PTBP3 knockdown in H520 and H1703 cells. Besides, CDC25A, one of the most crucial cell cycle regulators was also decreased in protein level (Fig. 4E, F). We performed a correlation analysis of PTBP3 with these three gene in the TCGA LUSC dataset. Results showed that the expression of CDC25A Cyclin A2 and CDK2 was positively correlated with PTBP3 (Additional file 4: Fig. S3A, B, C).

## Discussion

Polypyrimidine tract-binding protein (PTBP) preferentially binds to polypyrimidine-rich stretches of RNA, including PTBP1 PTBP2 and PTBP3. It functions mainly in splicing and can shuttle between the cytoplasm and the nucleus [16]. The functions of PTBP1 and PTBP2 have been well studied, whereas the role of PTBP3 has only been explored in recent years. Recently, some researchers have showed that PTBP3 was overexpressed and functioned as an oncogene in breast, gastric and hepatocellular cancer [13, 14, 17]. PTBP3 may play an emerging role in various cancer progressions. PTBP3 has previously been reported to promote metastasis in NSCLC and have no effect on cell proliferation [2]. Unfortunately, the authors did not break down the study into pathological types. Therefore, we were interested in what is the effect of PTBP3 in different pathological types of NSCLC.

One of our first major findings of our study was that the mRNA level of PTBP3 was dramatically higher both in LUAD and LUSC patients based on TCGA dataset. However, survival analyses based on TCGA databases showed that higher LUSC expression was associated with poor patient outcomes and was not significant difference in LUAD. In addition, the mRNA and protein level of PTBP3 was overexpression in our own clinical tumor tissues compared to normal tissues. We further investigated the relationship between PTBP3 and clinicopathological characteristics of LUSC and verified that higher PTBP3 expression resulted in larger tumor size than lower PTBP3 expression. These findings suggesting that PTBP3 may be involved in the development and progression of LUSC. In our study we investigated the roles of PTBP3 and provided evidence of potential molecular mechanism in LUSC.

To further understand the biological function of PTBP3 in LUSC processions, we knockdown the expression of PTBP3 in two LUSC cell lines (H520 and H1703) using a lentivirus-mediated shRNA. Our result our results showed that PTBP3 knockdown decreased tumor cell growth in vitro and vivo. These results indicated oncogenic activity of PTBP3 in LUSC procession.

To clarify the molecular mechanism of PTBP3 in LUSC tumorigenesis, RNA-seq analysis was used to explore enriched pathway in sh-PTBP3 vs sh-NC cells. The GO terms data indicated that PTBP3 involved in cell division processes, thus implying the effects of PTBP3 on cell proliferation. KEGG pathway and GSEA analysis showed that cell cycle also enriched in PTBP3 high expression phenotype. As we known, Dysregulation of cell cycle is closely related to cell proliferation [18]. Those all suggested that PTBP3 involved in the regulation of cell cycle to affect the occurrence and development of LUSC cell proliferation. In addition, Previous studies revealed that downregulation of PTBP3 induced apoptosis and cell cycle arrest through p53 signaling [19] or through HDAC6-pAkt-TYMS signaling [20]. Results from cell cycle assays showed an S phase arrest in cell lines with PTBP3 knockdown. At the same time, the Cyclin A2 and CDK2 expression were decreased in PTBP3 knockdown cells. Cyclin A2 associated with CDK2 functions in the S phase of the cell cycle [21] One reason for persistence of the cells in S phase may be that they are delayed from entering M phase because Rb inhibits cyclin A and CDC2 expression [22]. From this paper, we can learn that inhibition of cyclin A2 expression can cause cell cycle progression arrested in S phase. Hence, we demonstrate that PTBP3 regulates LUSC cell lines proliferation by regulating the S phase of the cell cycle. Recognized as a dual specific phosphatase, CDC25A contributes to kinase stimulation in conjunction with cyclin in cell cycle progression. CDC25A has an influence on mitosis, proliferation as well as the cell cycle progression at the S and G1 stages [23]. Some previous study indicated that CDC25A was related to cell cycle and the overexpression of CDC25A often occurred before neuron death [24]. The upregulation of CDC25A expression would lead to the growth of tumor in squamous cell carcinoma [25]. Besides, it has been illustrated that cyclin A/CDK2 is necessary for cells progression through the S phase [26], and CDK2 is the primary downstream substrate of CDC25A which activates cyclin A/CDK2 complex [27]. Therefore, these results suggested downregulation of CDC25A and cyclin A/CDK2 signaling pathways are involved in S phase arrest. The CDC25A also verified that controlling the cell‐cycle pathway could influence cell progression [28]. In our study, we found that PTBP3 repressed proliferation and arrest S phase through regulating CDC25A expression. Unfortunately, we have not elucidated the underlying mechanism by which PTBP3 interacts with CDC25A, this part we need further investigate. In summary, we have shown that PTBP3 can influence expression of CDC25A, which in turn can regulate cell cycle distribution as well as death in LUSC cells. We therefore suggest that PTBP3 expression has potential as a marker of clinical outcome in LUSC. Its potential prognostic significance in LUSC.

## Conclusion

In summary, we first demonstrated that PTBP3 was upregulated in LUSC tissues compared with normal tissues. Functionally, PTBP3 knockdown reduced the growth of LUSC cells by regulating the expression of cell cycle molecules. However, further investigation was still needed to elucidate the PTBP3 mechanism for the progression of LUSC, which will help to better understanding tumor progression of LUSC and guide the development of therapeutic targets for LUSC.

## Supplementary Information

**Additional file 1: Table S1.** Primer sequences used for qRT-PCR.

**Additional file 1: Figure S1.**Time-dependent ROC curves of the prognostic PTBP3 in the TCGA LUSC dataset.

**Additional file 1: Figure S2.**RNA-sequence analysis of heatmap and GSEA analysis. A. Heatmap of RNA-sequence results in H520-sh-NC and H520-sh-PTBP3 samples (n = 3). Genes with fold change ≥| 1 | and P value < 0.05 are shown. (B, C) GSEA identifies PTBP3-related signaling pathways based on TCGA LUSC dataset.

**Additional file 1: Figure S3.** Correlation analysis of PTBP3 in the TCGA LUSC dataset. A. PTBP3 positively correlated with CDK2 in LUSC tissues. B. PTBP3 positively correlated with CDC25A in LUSC tissues. C. PTBP3 positively correlated with CCNA2 in LUSC tissues.

## Data Availability

The datasets used or analyzed during the current study are available from the corresponding author on reasonable request.

## Abbreviations

PTBP3: Polypyrimidine tract-binding protein 3
CDC25A: Cell division cycle 25A
LUSC: Lung squamous cell carcinoma
CCK-8: Cell counting kit 8
CDK2: Cyclin dependent kinase 2
CDKs: Cyclin-dependent kinases
NSCLC: Non-small cell lung cancer
LUAD: Lung adenocarcinoma
OS: Overall survivals
ROD1: Regulator of differentiation 1
ATCC: American type culture collection
FBS: Fetal bovine serum
shRNA: Short hairpin Ribonucleic Acid
NC: Negative control
WB: Western blot
qRT-PCR: Quantitative real-time Polymerase Chain Reaction
GAPHD: Glyceraldehyde-3-phosphate dehydrogenase
PMSF: Phenylmethylsulphonyl fluoride
IgG-HRP: Immunoglobulin G- Horseradish Peroxidase
IHC: Immunohistochemistry
H&E: Hematoxylin–eosin staining
ROC: Receiver operating characteristic
AUCs: Area under the curves
DEGs: Differentially expressed genes
GO: Gene ontology
MF: Molecular function
CC: Cellular components
BP: Biological processes
KEGG: Kyoto Encyclopedia of Genes and Genomes
DAVID: Database for Annotation, Visualization and Integrated Discovery
GSEA: Gene set enrichment analysis
TCGA: The Cancer Genome Atlas
