# Supplementary material for: PTBP3 regulates proliferation of lung squamous cell carcinoma cells via CDC25A‐mediated cell cycle progression
Source: Cancer Cell Int. 2022 Jan 11;22:19. doi: 10.1186/s12935-022-02448-7 (PMC8753890; doi:10.1186/s12935-022-02448-7)
Supplement: Supplementary file 1 — Additional file 1: Table S1. Primer sequences used for qRT-PCR. [file 12935_2022_2448_MOESM1_ESM.docx]

Supplementary Table 1

**The targeted sequences of shRNA vectors**

Sh-PTBP3 sequence（5’ to 3’）： GCTGTCAGTGCCGTCCAATCA

NC-PTBP3 sequence（5’ to 3’）： TTCTCCGAACGTGTCACGT

**Primer sequences used for qRT-PCR**

| GeneID | Sequence (5’- 3’) | |
| --- | --- | --- |
| PTBP3-F | TCTGGCATCATTAACGGACCT |  |
| PTBP3-R | GCGAACAGGGAGGTCTATCT |  |
| GAPDH-F | gatttggtcgtattgggcgc |  |
| GAPDH-R | ttcccgttctcagccttgac |  |
