# Supplementary figures and images for: PTBP3 regulates proliferation of lung squamous cell carcinoma cells via CDC25A‐mediated cell cycle progression
Source: Cancer Cell Int. 2022 Jan 11;22:19. doi: 10.1186/s12935-022-02448-7 (PMC8753890; doi:10.1186/s12935-022-02448-7)

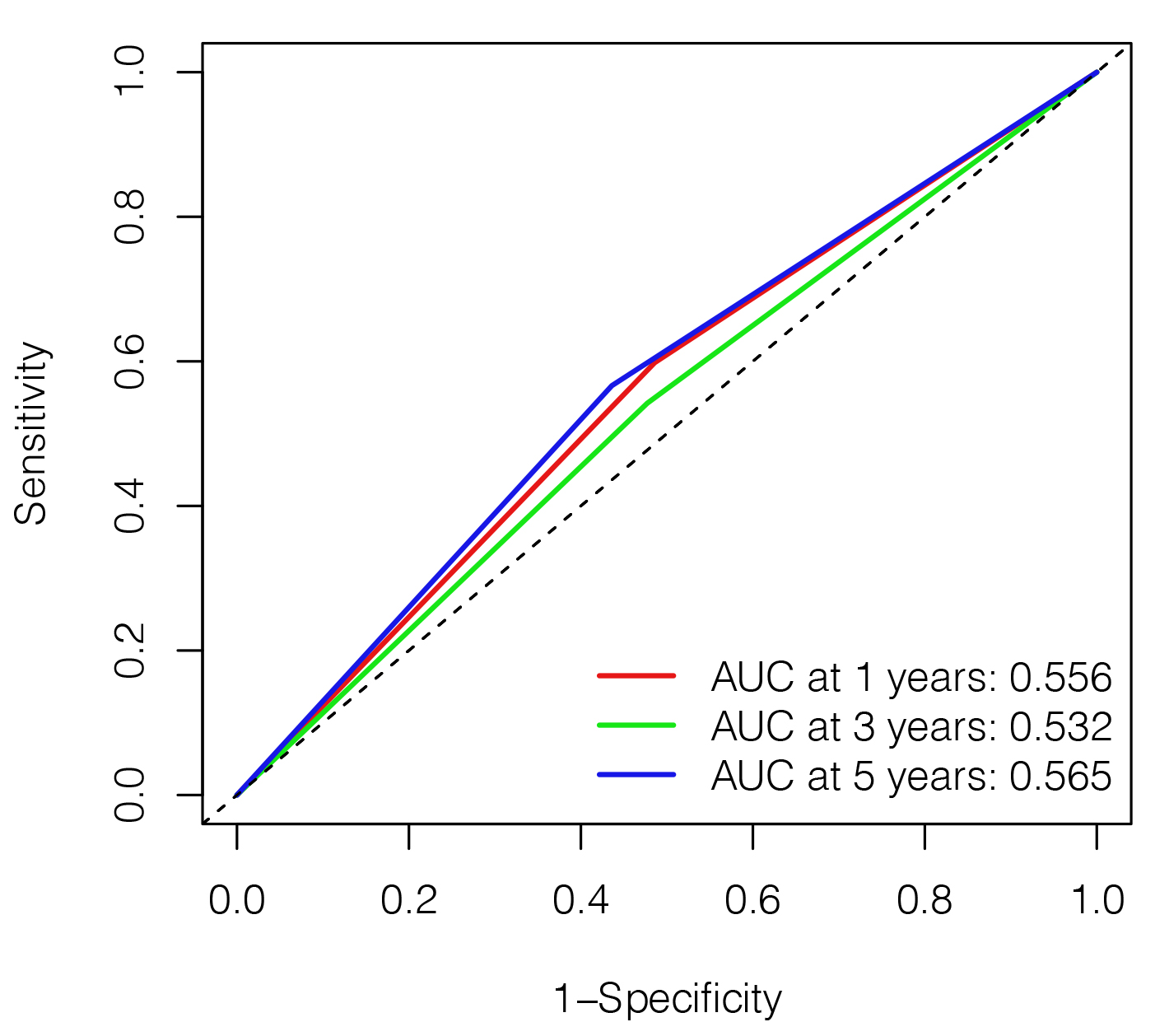

Supplement: Supplementary file 2 — Additional file 1: Figure S1.Time-dependent ROC curves of the prognostic PTBP3 in the TCGA LUSC dataset. [file 12935_2022_2448_MOESM2_ESM.jpg]

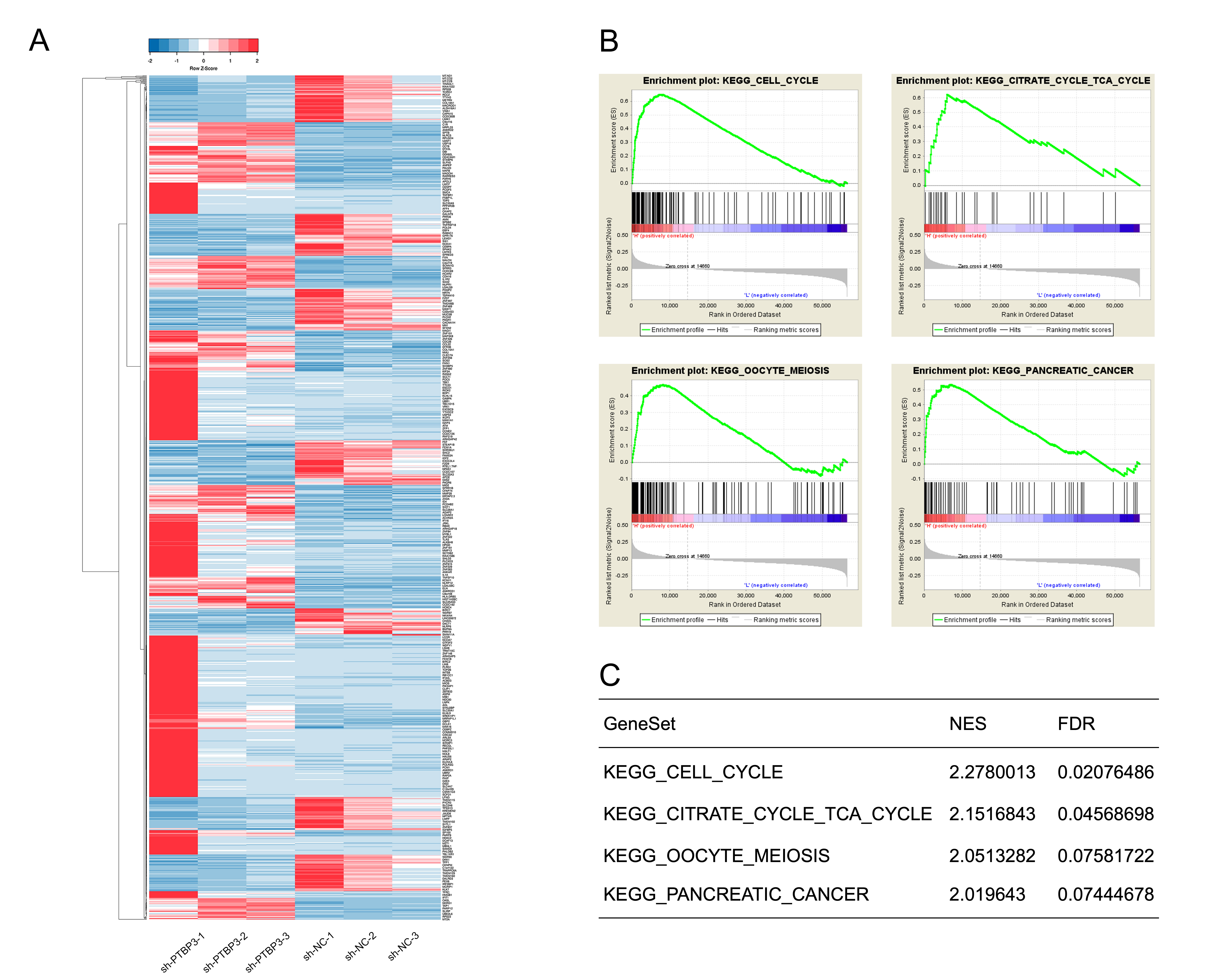

Supplement: Supplementary file 3 — Additional file 1: Figure S2.RNA-sequence analysis of heatmap and GSEA analysis. A. Heatmap of RNA-sequence results in H520-sh-NC and H520-sh-PTBP3 samples (n = 3). Genes with fold change ≥| 1 | and P value < 0.05 are shown. (B, C) GSEA identifies PTBP3-related signaling pathways based on TCGA LUSC dataset. [file 12935_2022_2448_MOESM3_ESM.jpg]

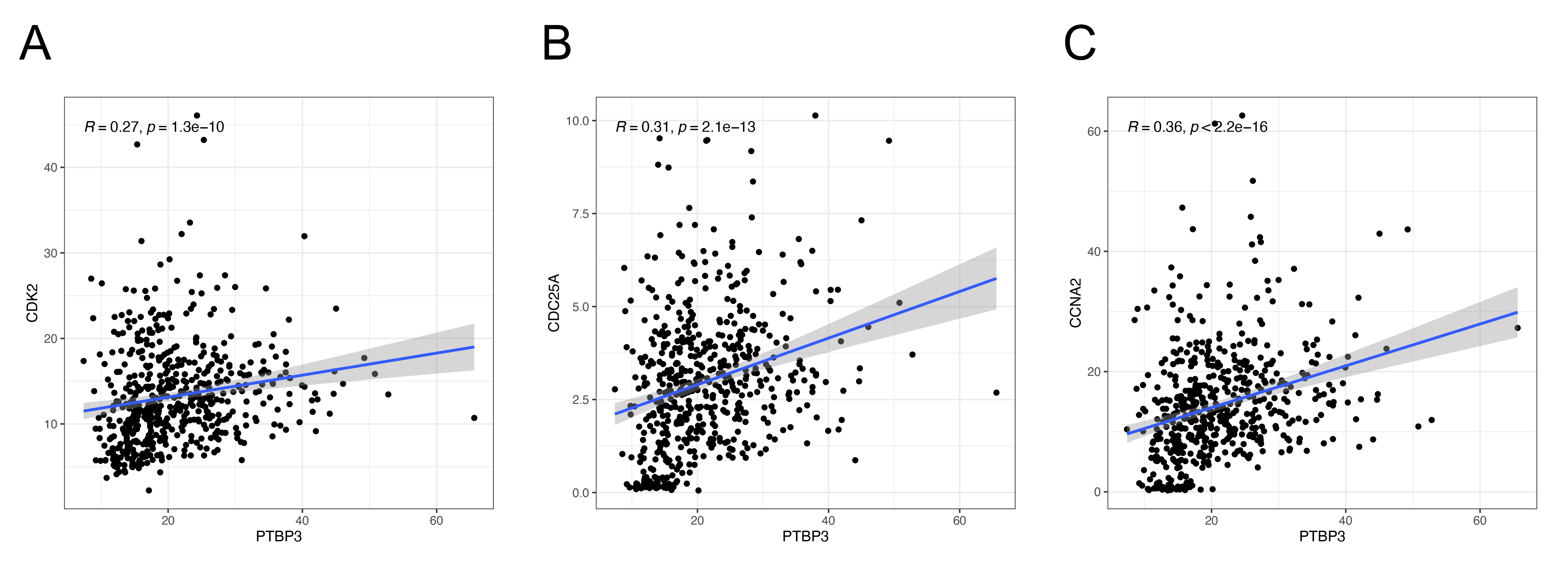

Supplement: Supplementary file 4 — Additional file 1: Figure S3. Correlation analysis of PTBP3 in the TCGA LUSC dataset. A. PTBP3 positively correlated with CDK2 in LUSC tissues. B. PTBP3 positively correlated with CDC25A in LUSC tissues. C. PTBP3 positively correlated with CCNA2 in LUSC tissues. [file 12935_2022_2448_MOESM4_ESM.jpg]
